# What determines childcare workers physical behaviours at work? An investigation of determinants at the institution, team, and worker levels in Danish day nurseries

**DOI:** 10.1093/annweh/wxaf016

**Published:** 2025-05-13

**Authors:** Christian Tolstrup Wester, Luiz Augusto Brusaca, Javier Palarea-Albaladejo, Stavros Kyriakidis, Anders Dreyer Frost, Andreas Holtermann, Charlotte Diana Nørregaard Rasmussen

**Affiliations:** Ergonomics and Musculoskeletal Health, The National Research Centre for the Working Environment, Lersø Parkallé 105, 2100 Copenhagen, Denmark; Ergonomics and Musculoskeletal Health, The National Research Centre for the Working Environment, Lersø Parkallé 105, 2100 Copenhagen, Denmark; Laboratory of Clinical and Occupational Kinesiology, Department of Physical Therapy, Federal University of Sao Carlos, Washington Luiz Road, km 235, SP310, 13565-905, São Carlos, São Paulo, Brazil; Department of Computer Science, Applied Mathematics & Statistics, University of Girona, C/ Universitat de Girona, 6 17003 - Girona, Spain; Ergonomics and Musculoskeletal Health, The National Research Centre for the Working Environment, Lersø Parkallé 105, 2100 Copenhagen, Denmark; Department of Sports Science and Clinical Biomechanics, University of Southern Denmark, Campusvej 55, 5230 Odense, Denmark; Ergonomics and Musculoskeletal Health, The National Research Centre for the Working Environment, Lersø Parkallé 105, 2100 Copenhagen, Denmark; Department of Sports Science and Clinical Biomechanics, University of Southern Denmark, Campusvej 55, 5230 Odense, Denmark; Ergonomics and Musculoskeletal Health, The National Research Centre for the Working Environment, Lersø Parkallé 105, 2100 Copenhagen, Denmark; Department of Sports Science and Clinical Biomechanics, University of Southern Denmark, Campusvej 55, 5230 Odense, Denmark; Ergonomics and Musculoskeletal Health, The National Research Centre for the Working Environment, Lersø Parkallé 105, 2100 Copenhagen, Denmark

**Keywords:** physical behaviours, childcare, accelerometry, organisational determinants, compositional data

## Abstract

**Objectives:**

The childcare sector faces several challenges such as high rates of sick leave, employee turnover, and pain. We know that the physical behaviours the workers do at work are important for their musculoskeletal health and sick leave. However, we lack knowledge of which workplace factors determine the physical behaviours of workers. Therefore, we aimed to investigate which organisational levels and factors of Danish day nurseries determine the physical behaviours at work of childcare workers.

**Methods:**

This cross-sectional study included 178 childcare workers from 73 teams and 16 day nurseries in Denmark. Workers were, on average, 36.5 years of age, mostly female (84%) and 57% were educated pedagogues. We measured physical behaviours (i.e. sedentary, light physical activity, and moderate-to-vigorous physical activity) during working hours using a thigh-worn accelerometer, and expressed them as isometric log-ratio coordinates for analysis according to compositional data analysis procedures. We examined 15 potential determinants of physical behaviours at work at three organisational levels: institutions (e.g. permanent-to-not-permanent staff ratio), teams (e.g. worker-to-child ratio), and workers (e.g. age, BMI, physical exertion). Variance component analysis identified the percentage contribution to the total variance of physical behaviours of each organisational level. Linear mixed models were used to investigate associations between determinants at each level and the physical behaviours.

**Results:**

The largest contribution to the total variance in childcare workers’ physical behaviours was observed at the worker level (95.5%), while team (2%) and institution (2.5%) levels contributed to only a minor extent. Two individual factors at the worker level—physical exertion (*P* < 0.01) and pain intensity (*P* = 0.01)—were significantly, but weakly associated with physical behaviours. Specifically, an increased physical exertion was associated with a 3.7% increase in moderate-to-vigorous physical activity (*P* = 0.019) and a 6% decrease in sedentary behaviour (*P* = 0.009), relative to the remaining behaviours. Also, an increased pain intensity was associated with a 3.6% decrease in moderate-to-vigorous physical activity (*P* = 0.008), relative to the remaining behaviours. No determinants at the institution and team levels were significantly associated with physical behaviours.

**Conclusions:**

In this study, worker level determines nearly all of the variability in physical behaviours while the institution and team levels only explain a little. That only two individual worker-level factors were weakly associated with physical behaviours indicates that other unmeasured worker-level factors are important determinants of the physical behaviours of childcare workers.

**What’s Important About This Paper?**

This study investigated which organisational levels and factors of Danish day nurseries determine workers’ physical behaviours at work. The results indicate that the worker level determines almost all variance in the physical behaviours among the workers. However, measured worker-level factors only play minor roles, which indicates that other unmeasured worker-level factors are important determinants for physical behaviours of childcare workers.

## Introduction

Denmark anticipates an increase of > 50 000 children aged 0 to 5 years by 2030. With a shortage of childcare workers (Kristoffersen and Christensen 2021; Lindeberg et al. 2023), there is an urgent need to improve the work-life expectancy of the workers. Moreover, many Danish day nurseries face challenges due to insufficient recruitment into the profession and a high employee turnover (Holtermann et al. 2020; Marckmann 2024).

Many childcare workers report musculoskeletal pain and high physical exertion at work (Rasmussen et al. 2020). Childcare workers in Denmark also report higher annual sick leave compared to other professions (NFA 2018a, 2018b; Flyvholm et al. 2019). These are considerable barriers to maintaining a sustainable work life in the profession (Coggon et al. 2020; Inoue et al. 2020).

Physical behaviour at work—such as high occupational physical activity—is known to be associated with impaired health (Coenen et al. 2018; Gupta et al. 2020a; Cillekens et al. 2022). Childcare work involves a range of physically demanding tasks, such as changing diapers, carrying children to comfort or for a nap, kneeling to engage at eye level, squatting to aid with clothing, and promoting physically active play (Schønheyder et al. 2024). These tasks require several strenuous physical behaviours, including lifting, kneeling, sitting on the floor, standing, walking at different paces, and occasionally running during play or when handling challenging situations (Holtermann et al. 2020).

However, studies have shown relatively large differences in the physical behaviours between childcare workers (Ward et al. 2018; Holtermann et al. 2020). As such, while some childcare workers are sitting a lot at work, others have high levels of physically demanding activities, which can impair their health (Holtermann et al. 2020). This indicates a potential for prevention through implementing a more even distribution of the physical behaviours at work between the childcare workers. However, this requires knowledge of which factors at various levels of the childcare organisation determine the physical behaviours of the childcare workers.

In a day nursery, the different organisational levels include the institution, the teams, and the workers. At each level, specific determinants can influence how tasks are allocated, which may lead to differences in the physical behaviours among the workers.

For instance, in institutions with a higher ratio of non-permanent-to-permanent employees, there may be larger differences in physical behaviours among the workers. Permanent employees may generally better know the daily routines, the organisation of the work and be more experienced in carrying out the childcare work, as compared to the non-permanent employees. As such, non-permanent employees may often be assigned simpler care tasks, such as actively playing with the children, while permanent employees may have more complex tasks such as providing care for children with special needs reflected in more sedentary behaviour or light physically active tasks. As such, institutions with a higher proportion of non-permanent employees may be more physically active compared to institutions with a lower proportion of non-permanent employees.

Similarly, in teams with lower worker-to-child ratios, there may be a greater focus on children’s basic care needs, leaving less time for physically active play, potentially leading to more time sitting during work compared to teams with a higher worker-to-child ratio. Furthermore, workers with musculoskeletal pain may not be able to do the most physically demanding tasks and are therefore more likely to be allocated tasks where they can sit more at work than colleagues without pain.

Because it is generally recommended to do preventive workplace interventions targeting higher organisational levels of workplaces (Aust et al. 2023), it is essential to know which organisational levels and determinants influence the physical behaviours of the workers. Current knowledge on organisational determinants of physical behaviours is limited (Stevens et al. 2021), and still unexamined in childcare work. Therefore, we aimed to investigate which organisational levels and factors of Danish day nurseries determine the physical behaviours at work of childcare workers.

## Methods

### Study design

This cross-sectional study is based on data from the TOY-project, which aimed to expand the body of research-based knowledge on physical work demands in childcare work (Rasmussen et al. 2018). Details regarding the study design, inclusion criteria and data collection are presented in the protocol paper (Rasmussen et al. 2018). In short, our data come from baseline measurements of childcare workers from 16 day nurseries. Twenty-nine eligible day nurseries in Copenhagen municipality accepted to participate. They all had children in the age group 0 to 3 years, and a minimum of nine childcare workers employed. However, resource restrictions limited the number of nurseries in the project to 16, which were randomly drawn from the sample of 29, and balanced by size (Rasmussen et al. 2018). Participation in the project was decided at the nursery institution level, but each individual childcare worker decided on participation in the evaluation of the project. No incentive for participation was given. The childcare workers were informed of the general aims of the study and gave written consent to participate. All procedures were performed according to the declaration of Helsinki. The study was approved by the Danish Ethics Committee to be approved via the National Research Centre for the Working Environments authorisation for low-risk non-invasive studies on healthy consenting adults, and does not need further reports to the local ethics committee (reference number 16048606). The study is registered in the ISRCTN Registry (ISRCTN10928313).

### Study population

A total of 220 childcare workers from 16 day nursery institutions consented to participate in the study. Of these, 212 (96.3%) workers had baseline data on at least one of the included determinants (e.g. age, sex, BMI, influence at work). Moreover, 178 of the childcare workers (80.9%) had valid accelerometer measurements of physical behaviours (see below) and were therefore included in this study.

### Accelerometer measurements of physical behaviours (outcome)

Physical behaviours were monitored using a thigh-worn triaxial AX3 accelerometer (3-Axis Logging Accelerometer; Axivity Ltd, Newcastle upon Tyne, UK), continuously for 24-h a day for up to 5 workdays. Note that previous studies suggest that about 4 days of measurements can be deemed enough to obtain valid measurements of the physical behaviours of individuals (Kocherginsky et al. 2017). Simultaneously, using a diary, workers were asked to record the time they ‘woke up’, ‘arrived at work’, ‘left from work’, ‘went to bed to sleep’, and if the device was detached. The accelerometer data were downloaded using the manufacturer’s software (OMGUI Version 1.0.0.30; Axivity Ltd, Newcastle upon Tyne, UK) and processed using a custom-made MATLAB program (*Acti4*; The National Research Centre for the Working Environment, Copenhagen, Denmark). The *Acti4* software classifies different postures and activities with a confirmed good validity, and high sensitivity and specificity (Skotte et al. 2014; Stemland et al. 2015).

For this study, the postures and activities were merged into three categories, i.e. physical behaviours: (i) sedentary behaviour (SB; lying, sitting), (ii) light physical activity (LPA; standing still, moving and slow walking), and (iii) moderate-to-vigorous physical activity (MVPA; fast walking, climbing stairs, running, cycling, and rowing). According to the purpose of the study, only working hours were used for further analysis and considered valid if they contained at least 4 h of measurements per day, in line with previous studies (Jørgensen et al. 2019).

### Determinants at the day nursery institution and team levels (exposures)

Determinants at the institution and team levels were collected by questionnaire from institution leaders.

*Institution-level* determinants were ‘not-permanent-to-permanent staff ratio’, and a variable indicating whether the institution was public or private. Information on the institution being public or private is based on workplace records. The not-permanent-to-permanent staff ratio variable was created through questions on the total number of permanently employed (‘How many permanent employees are in the day nursery institution?’) and not-permanently employed (‘How many not-permanent employees are in the institution, e.g. substitutes, students/interns?’) workers in the institution.

*Team-level* determinants were total ‘number of children’ in each team based on the question ‘How many children are in team number 1?’ (and accordingly for teams 2, 3, etc.), and the ‘worker-to-child ratio’. The worker-to-child ratio was calculated through information on which children and workers belonged to which respective team.

### Determinants at the worker level (exposures)

*Worker-level* determinants were collected by questionnaire or health assessments.

*Demographic and work environment* determinants included age (years), sex (male or female), weekly work hours, and job type (pedagogue/non-pedagogue). Job type was defined by being pedagogue if having a 3.5-year pedagogue education and non-pedagogue if having a shorter pedagogue-related education or no relevant education.

*Health*-related determinants included body mass index (BMI, kg/m^2^; calculated from the measured height and body weight obtained at a baseline health check), self-rated physical exertion measured on a 0 to 10 Likert scale (Borg 1982) and three measures of musculoskeletal pain (MSP). Measures of MSP were (i) ‘maximal pain intensity’ [0 to 10 on a numeric rating scale (NRS)] defined as the highest reported pain intensity in any one of eight body regions (lower back, neck, shoulders, knees, elbows, hands, hips, and feet/ankles), (ii) ‘pain regions’ (calculated as number of pain regions with an episode of pain (defined as > 1 day with pain in a body region and with an intensity of ≥ 3 on the NRS)), and (iii) ‘pain interference at work’ (days in the previous 4 weeks with pain that limits the ability to do the work) (Rasmussen et al. 2020).

*Psychosocial* determinants comprised two variables from the Danish Psychosocial Work Environment Questionnaire (DPQ): ‘Influence at work’ (e.g. ‘Do you have any influence on how you carry out your work tasks?’) and ‘Social support from colleagues’ (e.g. ‘Can you get practical help with your work from colleagues if you need it?’). Each of the two variables was based on four items reported on a 5-point Likert scale, which were subsequently converted into a 0 to 100 scale, with 0 representing, e.g. 'no influence on work tasks' (Clausen et al. 2019).

### Statistical analyses

All statistical analyses reported in this section were conducted on the R system for statistical computing v4.3.1 (R Core Team 2023); using the packages coda.base (Comas-Cufí 2023) and ggtern (Hamilton and Ferry 2018) for compositional data operations and visualisation, VCA for variance component analysis (Schuetzenmeister and Dufey 2022), and lme4 (Bates et al. 2015) for linear mixed modelling (Harrison et al. 2018). Statistical significance was generally concluded at the usual 5% significance level.

#### Compositional approach to the analysis of physical behaviours.

Time spent on physical behaviours are inherently co-dependent and constrained variables. They refer to fractions to a total time shared within,s e.g. a workday, meaning that more time can be spent in one physical behaviour only at the cost of reducing time in one or more of the other physical behaviours. In this setting, as the data are relative, a compositional data analysis (CoDA) approach is recommended for joint processing and statistical analysis (Pawlowsky-Glahn et al. 2015). This approach uses a log-ratio coordinate representation of the data to account for their special nature and to avoid issues with conventional analysis. Isometric log-ratio (ilr) coordinates have been widely adopted in time-use epidemiology (Chastin et al. 2015; Dumuid et al. 2020). For a description of the implementation of CoDA in occupational research, see, e.g. Gupta et al. (2020b).

The three-part work compositions of physical behaviours (SB, LPA, and MVPA) were transformed into two ilr coordinates using a sequential binary partition (Pawlowsky-Glahn et al. 2015; Dumuid et al. 2018).

The basic ilr coordinates used in this study were thus defined as:


$$\begin{array}{l} ilr_{1}=\sqrt{\frac{2}{\text{3}}}\ln\frac{SB}{{\left(LPA\cdot MVPA\right)}^{1/2}}andilr_{2}=\sqrt{\frac{1}{\text{2}}}\ln\frac{LPA}{MVPA}, \end{array}$$

where $ilr_{1}$ represents the contrast, or trade-off, of time between sedentary behaviours and the averaged (geometric mean) time spent in light physical activity and moderate-to-vigorous physical activity and, analogously, $ilr_{2}$ indicates time spent in LPA relative to MVPA. These unconstrained log-ratios are compatible with ordinary statistical analysis and models, and results can be translated back into the space of the original composition by back-transforming the ilr coordinates (Pawlowsky-Glahn et al. 2015).

#### Variance components analysis.

Firstly, we investigated which organisational *levels* (institution, team, and worker) of Danish day nurseries explained physical behaviours of childcare workers. As such, with the objective of learning about the hierarchy of contributions to the variability of the physical behaviours composition between childcare workers, a linear mixed model (LMM) was fitted by restricted maximum likelihood to the ilr coordinates $ilr_{1}$ and $ilr_{2}$, jointly, with ‘childcare institutions’ and ‘teams within the institutions’ included as nested random effects. Variance components were estimated by the algorithm given by Searle et al. (1992).

#### Associations between determinants at institution, team, and worker level and physical behaviours.

Secondly, we investigated which organisational factors of Danish day nurseries were associated with the physical behaviours of childcare workers. Associations between determinants at the institution, team, and worker levels, and the physical behaviours of the workers were investigated using LMMs. Our primary model (Model 1) was fitted to the whole composition as represented by the two ilr coordinates above, including all determinants as fixed effects and ‘childcare institutions’ and ‘teams within the institutions’ as nested random effects.

In a second analysis, we explored how organisational factors would relate to the dominance of each physical behaviour against the others, i.e. how each factor was associated with, respectively, more or less time in SB, LPA, and MVPA relative to the average time spent on the other behaviours (see Supplementary Table S1, Model 2a–c). This analysis involved three LMMs with the same overall structure as Model 1 but rotating (pivoting) each physical behaviour to be placed once at the first position in the ilr coordinate system. Thus, we followed here the pivot coordinate approach (Chastin *et al.* 2015; Dumuid *et al.* 2020), and used such first coordinate as the response variable in each model. To facilitate interpretation, the results were expressed as percentage change similar to the procedure used in Burge et al. (2021) (see further details of this calculation in Supplementary File S2 and Supplementary Table S2).

Finally, ternary plots were created to visualise the relationship between estimated compositions (SB, LPA, and MVPA) and the statistically significant determinants resulting from the Model 1 fit. To do so, the estimates obtained from the modelling, in terms of the coordinates [$ilr_{1}$, $ilr_{2}$], were translated back into the original space of three-part compositions of physical behaviours, and thereby expressed as fractions of the total work time by applying the corresponding inverse ilr coordinate transformation (Pawlowsky-Glahn et al. 2015).

## Results

### Descriptive summary of the study population

Characteristics of the sample are provided in Table 1. Workers were, on average, 36.5 (SD 11.7) years of age, mostly female (84%) and 57% were educated pedagogues. Average physical exertion level was nearly 6 out of 10 and maximal pain intensity level was nearly 6 out of 10.

**Table 1.** Characteristics of workers, teams, and institutions.

*Determinants*	Mean (SD)	*N* (%)
**Worker (*n* = 178)** Demographics and work environment		
Age	36.5 (11.7)	
Sex (male/female)		
Female		150 (84.3%)
Male		23 (15.7%)
Work hours	35.0 (2.6)	
Job type		
Pedagogue		101 (56.7%)
Non-pedagogue		77 (43.3%)
Physical health		
BMI	25.3 (5.3)	
Physical exertion (0–10)	5.9 (1.8)	
Number of pain regions (0–8)	2.5 (1.8)	
Max pain intensity min 1 region (0–10)	5.6 (2.5)	
Pain interference at work (0–28 days)	3.5 (6.6)	
Psychosocial determinants		
Influence work tasks (0–100)	69.8 (14.9)	
Support from colleagues (0–100)	70.8 (14.3)	
**Team (*n* = 73)**		
Number of children	12.0 (0.9)	
Worker-to-child ratio	0.3 (0.09)	
**Institution (*n* = 16)**		
Number of workers	20.3 (8.3)	
Private or public institution		
Public		10 (62.5%)^1^
Private		6 (37.5%)^1^
Not-permanent-to-permanent staff ratio	0.3 (0.2)	
Number of not-permanent workers	4.6 (3.7)	
Number of permanent workers	15.8 (5.3)	

^1^Ten public institutions with 140 employees (78.7%), six private institutions with 32 employees (21.3%).

Physical behaviours of the workers were measured averagely 6.61 h per day (geometric mean), and between 1 and 5 days (3.3 days on average) using thigh-worn accelerometry. Workers spent on average 47.7% of the workday sedentary, 40% in LPA, and 12.3% in MVPA (see Table 2; using geometric means for consistency with the compositional nature and closing, or scaling, to have it in percentage).

**Table 2.** Average distribution of physical behaviours during work among 178 childcare workers.

Physical behaviours	Average hours (geometric means)	In %
Sedentary behaviour	3.17	47.67
Light physical activity	2.66	40.00
Moderate-to-vigorous physical activity	0.82	12.33

Each institution had averagely 20 employed workers, however, with large variations and averagely around 16 permanently employed, and 4 not-permanently employed workers. Teams in the institutions had an average of 12 children, and averagely 4 children per worker (Table 1).

### Organisational levels and variance in physical behaviours

The variation in physical behaviours primarily originated at the worker level (variance component estimate = 0.079), with a relative contribution to the total variance of 95.5% (Table 3). Amongst the team and institution levels, the contribution to total variance was 2.0% and 2.5%, respectively. Considering only the team and institution levels, the variance was distributed as 45.7% for the team level and 54.3% for the institution level.

**Table 3.** Variance components analysis of physical behaviours at different organisational levels of childcare institutions (178 workers, 73 teams, and 16 institutions).

Level	Estimate	% Contribution
Worker	0.079	95.5
Team	0.0017	2.0
Institution	0.0021	2.5
Total	0.083	100

### Organisational determinants of physical behaviours

When investigating which organisational factors related to the physical behaviours composition as a whole (Model 1), we found no statistically significant associations for the institution- or team-level determinants. At the worker level, statistically significant associations were found for physical exertion (*P* = 0.008) and pain intensity (*P* = 0.010) (see Table 4).

**Table 4.** Associations between the physical behaviour composition and determinants at institution, team, and worker levels.

	Model 1 (N = 155*)	
*Determinants*	β (95% CI)	*p*
Worker level		
Demographics/work environment		
Age	0.00 (−0.00–0.00)	0.231
Sex [ref. male]	−0.01 (−0.12–0.10)	0.818
Work hours	−0.00 (−0.02–0.01)	0.562
Job type [non-pedagogue]	−0.04 (−0.11–0.03)	0.243
Physical health		
BMI	−0.01 (−0.01–0.00)	0.078
Physical exertion	−0.03 (−0.05–−0.01)	**0.008**
Pain regions (pain intensity > 3)	−0.00 (−0.03–0.02)	0.807
Max pain intensity	0.03 (0.01–0.04)	**0.010**
Pain interference at work	−0.00 (−0.01–0.00)	0.986
Psycho-social determinants		
Influence on tasks	−0.00 (−0.00–0.00)	0.509
Support from colleagues	0.00 (−0.00–0.00)	0.324
Team level		
Number of children	0.02 (−0.03–0.06)	0.473
Worker-to-child ratio	−0.36 (−1.01–0.30)	0.283
Institution level		
Private/public institution [ref. private]	−0.08 (−0.17–0.01)	0.082
Not-permanent-to-permanent staff ratio	−0.08 (−0.29–0.12)	0.427

^*^155 workers, 69 teams, 16 institutions. CI = Confidence interval, β = beta coefficient, *p* = *P*-value. Physical behaviour composition components: sedentary, light physical activity, and moderate-to vigorous physical activity.

In our second analysis, investigating how organisational factors were related to the dominance of each behaviour relative to the average of the others (as specified through pivot coordinates), no statistically significant associations were found for the institution- or team-level determinants for any of the physical behaviours (see Supplementary Table S1, Model 2a–c). At the worker level, we found that an increase in physical exertion was associated with a 3.7% increase in MVPA (*P* = 0.019), and a 6% decrease in SB (*P* = 0.009), relative to the remaining behaviours in each case. Increased pain intensity was associated with a 3.6% decrease in MVPA (*P* = 0.008), and an increased BMI was associated with a 1.2% increase in MVPA (*P* = 0.022) relative to the remaining behaviours (see further details of the percentage calculation in Supplementary File S2 and Supplementary Table S2).

**Fig. 1.**
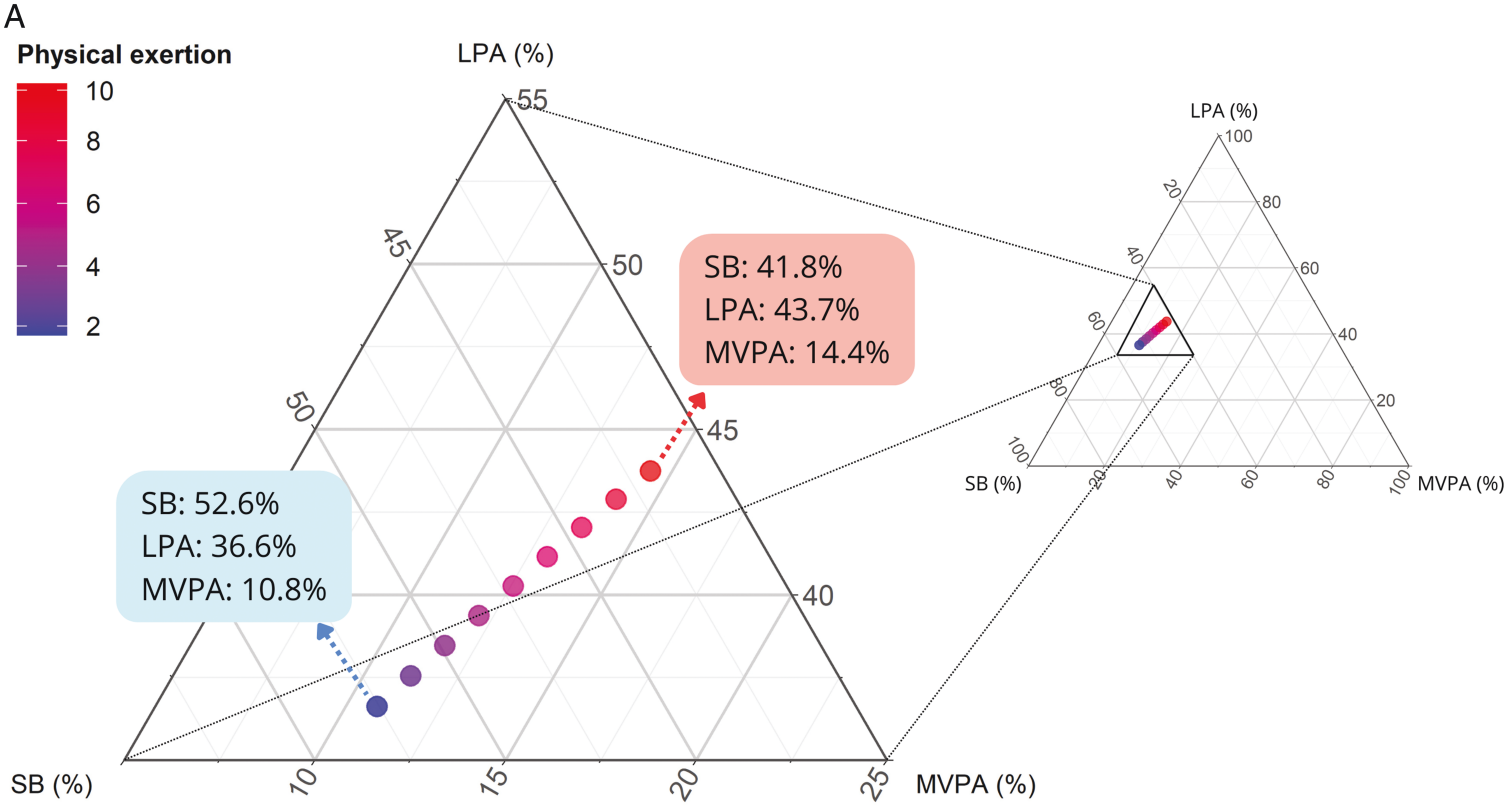

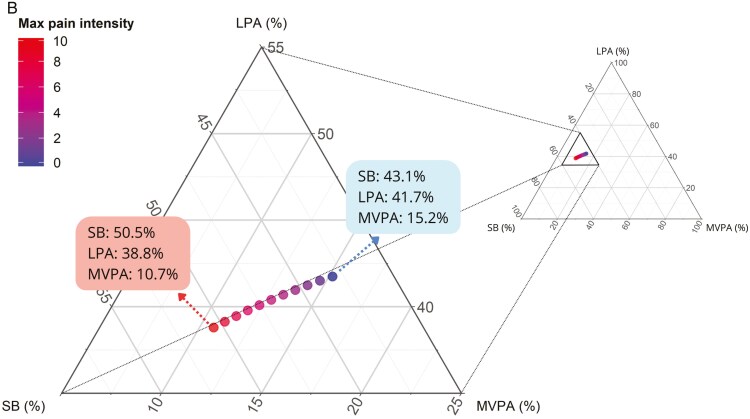
Physical behaviour compositions - sedentary behaviour (SB), light physical activity (LPA) and moderate-to-vigorous physical activity (MVPA) - predicted by linear mixed modelling for workers reported a) physical exertion ranging in [2, 10] on a 0 to 10 scale and b) maximal pain intensity ranging in [0, 10] on a 0 to 10 scale.

Figure 1a and b presents ternary diagrams showing trends in physical behaviour based on changes in physical exertion and pain intensity, respectively. Figure 1a illustrates that as physical exertion increases, there is a shift towards more time spent in LPA (36.6% to 43.7%) and MVPA (10.8% to 14.4%) at the expense of time spent in SB (52.6% to 41.8%). Figure 1b illustrates that as pain intensity increases, there is a shift towards more time spent in SB (43.1% to 50.5%) and a reduction in the time spent in LPA (41.7% to 38.8%) and MVPA (15.2% to 10.7%).

## Discussion

This study aimed to investigate which organisational levels and factors of Danish day nurseries determine the physical behaviours at work of childcare workers. We investigated this across three levels of the organisation: institutions, teams, and workers. To our knowledge, this is the first study to investigate how organisational levels and determinants explain the variation in physical behaviours among childcare workers.

Overall, among the three organisational levels of the day nurseries we found that the worker level determined nearly all of the physical behaviours (95.5%), while the institution and team levels explained only a little. Moreover, while we found no associations for any of the team- and institution-level factors, two worker-level factors (physical exertion and pain intensity) were associated with differences in physical behaviours at work of the childcare workers. This finding aligns well with the finding that team and institution levels only explained a small fraction of the variance in physical behaviours.

The negligible influence of team and institution levels on the physical behaviours of the workers may be explained by similarities between the institutions in their organisational structures, and similarities between the teams in how to perform the work activities (i.e. tasks). For instance, although work activities vary between workers, the work tasks and activities between teams may be similar, involving approximately the same distribution of time in SB, LPA, and MVPA. Additionally, the childcare institutions included in our study were all located in Copenhagen municipality, which can be more homogeneous in terms of, e.g. number of children per pedagogue, norms or policies, compared to institutions in other cities or rural areas of Denmark.

At the institutional level, although not reaching statistical significance, a higher proportion of non-permanent staff relative to permanent staff was associated with a 30.8% decrease in sedentary behaviour and 41% increased light physical activity among workers. As such, the association showed notable effect sizes potentially of meaningful relevance, indicating that institutions with more non-permanent workers face higher physical activity at work. This could be due to non-permanent workers only being trained and assigned tasks requiring less formal education, such as actively playing with the children. While this is a likely explanation, we did not find any studies to confirm this. The higher physical activity at work among non-permanent workers may in turn lead to a more uneven distribution of work tasks in these institutions, with some workers having more physically strenuous work activities. However, as this is an observational study, we cannot determine whether this is a causal relationship. Thus, this should be further investigated in an interventional study design.

At the worker level, we found that two determinants were significantly associated with physical behaviours at work. Firstly, higher physical exertion correlated with more time spent in moderate-to-vigorous activities and less time in sedentary behaviour. This observation is consistent with previous findings among eldercare workers (Januario et al. 2020). Workers with higher physical exertion may be engaged in more physically strenuous tasks, such as lifting and carrying children, compared to more sedentary tasks, such as feeding the children or sitting on the floor and playing with the children. It could also be that workers with higher physical capacities are allocated to the most physically demanding tasks, as they may tolerate higher physical exertion levels compared to workers with a lower physical capacity. Thus, this result could be explained by reverse causality and by how the work tasks are allocated to the workers.

Secondly, higher pain intensity was associated with less time spent in moderate-to-vigorous activities during work hours. We believe the most likely explanation for this finding is that workers with pain are allocated or choose the less physically demanding work tasks, involving more time sitting compared to their colleagues. Thus, workers with little or no pain may generally be assigned work tasks involving higher physical activity levels. Future studies on childcare workers with larger samples should further explore whether the workers’ physical capabilities determine what role they fill in the day nurseries, and for instance, if non-permanent workers might have more strenuous tasks than permanent workers due to being markedly younger and therefore more physically capable.

### Practical implications

Although we found that the majority of the variance in physical behaviours at work is explained at the worker level, it is important to emphasise that this does not mean that workplace interventions to improve the physical demands of childcare workers should target the individual worker. This is supported by only two individual worker factors being weakly associated with the physical behaviours of the workers. However, when most variance in physical behaviours is found at the worker level, we believe that ‘unmeasured’ factors at the worker level are important determinants for the physical behaviours at work. One such unmeasured factor could be related to how work tasks are allocated between the workers within their teams. However, this assumption needs to be further investigated before being communicated and brought into practice. This could be assessed by collecting data on work schedules detailing specific tasks throughout the day or through direct workplace observations of task allocation.

While our study did not find associations between institution- or team-level factors and physical behaviours, it is likely that work planning and task allocation between workers within teams play a significant role in determining these behaviours. Moreover, given that the individual worker factors were not or only weakly associated with the physical behaviours, workplace interventions could instead focus on how the work tasks are planned and distributed between workers within teams to promote that as many workers as possible achieve a healthy balance of physical behaviours (Lerche et al. 2020). For example, interventions could aim for a more balanced distribution of tasks among workers, e.g. between permanent and non-permanent employees, so that no individual worker is consistently assigned a high proportion of sedentary or strenuous activities. Moreover, focus on team collaboration in physically demanding tasks may reduce physical strain on the individual worker and contribute to a healthier work environment (Lohne et al. 2022, 2024).

Currently, limited research has evaluated such interventions among childcare workers (Lund Rasmussen et al. 2022; Schmidt et al. 2024). To develop effective interventions that promote healthy physical behaviours at work among childcare workers, we recommend that future studies examine how factors such as planning and allocation of work tasks between the workers within their teams influence the physical behaviours of the workers.

In addition, this study suggests that attention should be paid to workers experiencing high levels of pain or physical exertion. Efforts should be made to achieve a balanced work activity pattern, where workers are involved in a variety of tasks, including sitting, standing, and walking, as well as actively playing with the children, to engage in the different physical behaviours throughout the workday.

### Strengths and limitations

The major strengths of this study are the use of accelerometers to measure physical behaviours over multiple days, and the multi-level design allowing us to account for the effects at the organisational levels we assessed (worker, team, and day nursery institution). Moreover, the use of a compositional data analysis approach is still limited within occupational health research (Gupta et al. 2020b). Only a few studies have investigated potential reasons for the variation in physical behaviours, i.e. having the composition of the physical behaviours as dependent variable (von Rosen 2023).

Amongst the limitations of the study, firstly, its cross-sectional nature limited the ability to infer causal relationships. Moreover, the relatively small sample size might have prevented the study from uncovering some meaningful associations or effects between the determinants and physical behaviours. However, as the literature is sparse on this topic, in our view, this is a relevant first study that contributes to understanding which organisational levels and factors of day nurseries are associated with the physical behaviours of the workers. Additionally, self-reported data, such as pain and physical exertion, may be subject to recall and social desirability bias, which could influence the results. However, self-reported measures, such as perceived physical exertion, remain valuable for capturing subjective experiences that objective measures (e.g. accelerometers) cannot assess. Furthermore, while our approach follows best practices for analysing compositional data, compositional data analysis is still evolving in the context of occupational physical behaviour research, and the lack of standardised measures in this field may affect comparability across studies. Finally, only a limited number of institutional and team-level factors were assessed, meaning that other potentially influential workplace characteristics, such as leadership style or institution size, were not accounted for.

## Conclusions

This study found that the worker level determines nearly all of the observed variability in physical behaviours in childcare workers during work hours, while the team and institution levels only explain a little. Only two individual worker factors were weakly associated with the physical behaviours, indicating that other unmeasured worker-level factors may be important determinants for physical behaviours. These unmeasured factors could be related to the allocation of work tasks between the workers within teams.

These findings may be useful in designing actions and policies to promote healthier compositions of physical behaviours of workers in the childcare sector.

## Supplementary material

Supplementary material is available at *Annals of Work Exposures and Health* online.

wxaf016_suppl_Supplementary_Material

## Data Availability

The datasets generated during and/or analysed during the current study are not publicly available in order to protect the anonymity of the participants and ensure that the GDPR is being followed; therefore, data sharing is not available in the current format. Please contact the corresponding author for further information.
