# Supplementary material for: What determines childcare workers physical behaviours at work? An investigation of determinants at the institution, team, and worker levels in Danish day nurseries
Source: Ann Work Expo Health. 2025 May 13;69(5):520–30. doi: 10.1093/annweh/wxaf016 (PMC12208360; doi:10.1093/annweh/wxaf016)
Supplement: wxaf016_suppl_Supplementary_Material [file wxaf016_suppl_supplementary_material.pdf]

Supplementary material

What determines childcare workers physical behaviours at work?
- An investigation of determinants at the institution, team, and worker levels in Danish day nurseries

Authors: Christian Tolstrup Wester[1], Luiz Augusto Brusaca[1,2], Javier Palarea-Albaladejo[3], Stavros Kyriakidis[1,4], Anders Dreyer Frost[1,4], Andreas Holtermann[1,4], Charlotte Diana Nørregaard Rasmussen[1]

[1]Department of Ergonomics and Musculoskeletal Health, The National Research Centre for the Working Environment, Lersø Parkallé 105, 2100 Copenhagen, Denmark
[2]Laboratory of Clinical and Occupational Kinesiology, Department of Physical Therapy, Federal University of Sao Carlos, Washington Luiz Road, km 235, SP310, 13565-905, São Carlos, São Paulo, Brazil
[3]Department of Computer Science, Applied Mathematics & Statistics, University of Girona, C/ Universitat de Girona, 6 17003 - Girona, Spain
[4] Department of Sports Science and Clinical Biomechanics, University of Southern Denmark, Campusvej 55, 5230 Odense, Denmark

Corresponding Author: Christian Tolstrup Wester, Department of Ergonomics and Musculoskeletal Health, The National Research Centre for the Working Environment, Lersø Parkallé 105, 2100 Copenhagen Ø, Denmark, Email: ctw@nfa.dk

Table S1. Associations between dominance of physical behaviours and potential determinants

	Model 2a ($ilr_1^{(SB)}$)		Model 2b ($ilr_1^{(LPA)}$)		Model 2c ($ilr_1^{(MVPA)}$)	
Determinants	β (95% CI)	p	β (95% CI)	p	β (95% CI)	p
**Worker** (N=155)						
Demographics/work environment						
Age	0.00 (-0.01 – 0.00)	0.944	0.00 (-0.00 – 0.01)	0.120	-0.00 (-0.01 – 0.00)	0.107
Sex [Male]	-0.11 (-0.29 – 0.07)	0.215	0.13 (-0.01 – 0.26)	0.071	-0.04 (-0.17 – 0.10)	0.594
Work hours	-0.02 (-0.04 – 0.01)	0.122	0.02 (-0.00 – 0.04)	0.073	0.00 (-0.02 – 0.02)	0.853
Job type [non-pedagogue]	-0.06 (-0.17 – 0.06)	0.317	0.01 (-0.09 – 0.09)	0.954	0.05 (-0.04 – 0.14)	0.248
Physical health						
BMI	0.00 (-0.01-0.01)	0.573	-0.01 (-0.01 – 0.00)	0.171	0.01 (0.00 – 0.02)	**0.022**
Physical exertion	-0.05 (-0.08 – -0.01)	**0.009**	0.02 (-0.01 – 0.04)	0.306	0.03 (0.01 – 0.06)	**0.019**
Pain regions	-0.00 (-0.04 – 0.04)	0.979	-0.00 (-0.04 – 0.03)	0.819	0.01 (-0.02 – 0.04)	0.722
Max pain intensity	0.03 (0.00 – 0.06)	0.054	0.00 (-0.02 – 0.03)	0.908	-0.03 (-0.06 – -0.01)	**0.008**
Pain interference work	-0.00 (-0.01 – 0.01)	0.603	0.00 (-0.01 – 0.01)	0.521	0.00 (-0.01 – 0.01)	0.844
Psycho-social determinants						
Influence tasks	-0.00 (-0.01 – 0.00)	0.089	0.00 (-0.00 – 0.01)	0.055	0.00 (-0.00 – 0.00)	0.935
Support colleagues	0.00 (-0.00 – 0.01)	0.058	-0.00 (-0.01 – 0.00)	0.079	-0.00 (-0.00 – 0.00)	0.658
**Team** (N=69)						
Number of children	-0.01 (-0.08 – 0.05)	0.695	0.05 (-0.01 – 0.11)	0.097	-0.04 (-0.09 – 0.02)	0.183
Worker-to-child ratio	-0.79 (-1.84 – 0.25)	0.136	1.64 (-1.67 – 4.94)	0.255	0.28 (-0.60 – 1.15)	0.534
**Institution** (N=16)						
Private/public [Private]	-0.10 (-0.24 – 0.05)	0.185	0.36 (-0.27 – 0.98)	0.930	0.10 (-0.03 – 0.23)	0.140
Not-permanent-to-permanent staff ratio	-0.30 (-0.63 – 0.04)	0.083	0.28 (-0.04 – 0.59)	0.094	0.02 (-0.28 – 0.31)	0.905

β stands for the unstandardized coefficient for the determinant in each of the models with the first pivot coordinate (ilr_1) set as response variable. SB: sedentary behaviour, LPA: light physical activity, MVPA: moderate-to vigorous physical activity. Statistical significance concluded for $p < 0.05$.

Supplementary file S2: Description of rotation of the behaviours in the compositional analysis

Three models were defined based on the physical activity behaviours placed in the numerator of the ilr_1 coordinate, which was used as the single response variable in the LMM formula:

- Model 2a: SB relative to the remaining behaviours (LPA and MVPA).
- Model 2b: LPA relative to the remaining behaviours (SB and MVPA).
- Model 2c: MVPA relative to the remaining behaviours (SB and LPA).

The corresponding ilr_1 coordinates are:

$$ilr_1^{(SB)} = \sqrt{\frac{2}{3}} \ln \frac{SB}{(LPA \cdot MVPA)^{1/2}},\ ilr_1^{(LPA)} = \sqrt{\frac{2}{3}} \ln \frac{LPA}{(SB \cdot MVPA)^{1/2}} \text{ and } ilr_1^{(MVPA)} = \sqrt{\frac{2}{3}} \ln \frac{MVPA}{(SB \cdot LPA)^{1/2}}.$$

(Note that a superscript indicating the reference behaviour has been added to facilitate interpretation).

The results are presented in terms of how a unitary increase in each of the statistically significant determinants, while keeping other variables constant, affects the relative dominance of specific physical behaviours compared to the average of the remaining behaviours. Moreover, we calculated the percentage change based on a specific formula in a recent paper from Burge and colleagues (Burge et al. 2021).

Thus, the following specific formula was used for the interpretation of regression coefficients in compositional models as multiplicative changes in the relative dominance of a behavior:

$$\delta = 2^{\beta *},$$

with β* = $log_2\ (e) \sqrt{\frac{D}{D-1}} \times \beta$, and where:

- δ = multiplicative change of the dominance of one behavior (relative to the average of the others) due to a one unit increase in the exposure (e.g. physical exertion), keep any other covariates constant.
- *e* = mathematical constant. Used to change from original natural base of the logarithm to base 2.
- *D* = number of components of the composition (in the case of the current study this is 3 (SB, LPA and MVPA).
- β = original regression model coefficient estimate (see Table A1, e.g. this is -0.05 for the example of the result for physical exertion and SB).

For example, using results in Table A1 for physical exertion and SB, the calculation would be

$$\beta^* = log_2(e) \sqrt{\frac{3}{3-1}} \times (-0.05) = -0.0883$$

and

$$\delta = 2^{-0.0883} = 0.9406$$

Then:

- Relative change (Δ): $\Delta = |\ 1 - 0.9406\ | = 0.0594$
- Expressed in percentage: $\Delta\% = 100 * \Delta = 5.94\%$

Table S2. Compositional regression model coefficients and calculation of multiplicative changes in the relative dominance of the physical activity behaviours.

Variable	β	δ^{1}	Δ^{2}	Δ-%	Direction
**BMI**<br>Model 2c (ilr[1](MVPA)	0.01	1.01	0.012	1.2%	Increase
**Physical exertion**<br>Model 2a (ilr[1](SB))	-0.05	0.94	0.059	5.9%	Reduction
**Physical exertion**<br>Model 2c (ilr[1](MVPA))	0.03	1.04	0.037	3.7%	Increase
**Max pain intensity**<br>Model 2c (ilr[1](MVPA))	-0.03	0.96	0.036	3.6%	Reduction

[1] Multiplicative change, [2] Relative change
